# Genetic diversity and population structure of date palms (*Phoenix dactylifera* L.) in Ethiopia using microsatellite markers

**DOI:** 10.1186/s43141-021-00168-5

**Published:** 2021-05-07

**Authors:** Workia Ahmed, Tileye Feyissa, Kassahun Tesfaye, Sumaira Farrakh

**Affiliations:** 1 Institute of Biotechnology, Addis Ababa University, Addis Ababa, Ethiopia; 2 Department of Biology, Wolkite University, Wolkite, Ethiopia; 3 Ethiopian Biotechnology Institute (EBTi), Ministry of Science and Technology (MoST), Addis Ababa, Ethiopia; 4 Department of Biosciences, COMSATS University of Islamabad, Islamabad, Pakistan

**Keywords:** Date palm (*Phoenix dactylifera* L.), Genetic diversity, Ethiopia, Microsatellites, Polymorphism, Population structure

## Abstract

**Background:**

Date palm tree (*Phoenix dactylifera* L.) is a perennial monocotyledonous plant belonging to the Arecaceae family, a special plant with extraordinary nature that gives eminent contributions in agricultural sustainability and huge socio-economic value in many countries of the world including Ethiopia. Evaluation of genetic diversity across date palms at DNA level is very important for breeding and conservation. The result of this study could help to design for genetic improvement and develop germplasm introduction programmes of date palms mainly in Ethiopia.

**Results:**

In this study, 124 date palm genotypes were collected, and 10 polymorphic microsatellite markers were used. Among 10 microsatellites, MPdCIR085 and MPdCIR093 loci showed the highest value of observed and expected heterozygosity, maximum number of alleles, and highest polymorphic information content values. A total of 112 number of alleles were found, and the mean number of major allele frequency was 0.26, with numbers ranging from 0.155 (MPdCIR085) to 0.374 (MPdCIR016); effective number of alleles with a mean value of 6.61, private alleles ranged from 0.0 to 0.65; observed heterozygosity ranged from 0.355 to 0.726; expected heterozygosity varied from 0.669 to 0.906, polymorphic information content with a mean value of 0.809; fixation index individuals relative to subpopulations ranged from 0.028 for locus MPdCIR032 to 0.548 for locus MPdCIR025, while subpopulations relative to total population value ranged from − 0.007 (MPdCIR070) to 0.891 (MPdCIR015). All nine accesstions, neighbour-joining clustering analysis, based on dissimilarity coefficient values were grouped into five major categories; in population STRUCTURE analysis at highest K value, three groups were formed, whereas DAPC separated date palm genotypes into eight clusters using the first two linear discriminants. Principal coordinate analysis was explained, with a 17.33% total of variation in all populations. Generally, the result of this study revealed the presence of allele variations and high heterozygosity (> 0.7) in date palm genotypes.

**Conclusions:**

Microsatellites (SSR) are one of the most preferable molecular markers for the study of genetic diversity and population structure of plants. In this study, we found the presence of genetic variations of date palm genotypes in Ethiopia; therefore, these genetic variations of date palms is important for crop improvement and conservation programmes; also, it will be used as sources of information to national and international genbanks.

**Supplementary Information:**

The online version contains supplementary material available at 10.1186/s43141-021-00168-5.

## Background

Date palm tree (*Phoenix dactylifera* L.) is a diploid plant with 2*n* = 36 chromosome number, a perennial monocotyledonous plant belonging to the Arecaceae family [1, 2]. It is one of the oldest known fruit-bearing tree crops with extensive cultivation and utilization in North Africa and Middle East for at least 5000 years and believed to have originated in Mesopotamia [2–5]. Date palms have a great socio-economic impact and an eminent contribution in agricultural sustainability in many arid and semiarid parts of the world [1, 6]. It is a multipurpose tree having food, industrial, commercial, medicinal and ornamental values [7, 8].

Date fruits have high nutritional value and contain about 70% sugar, essential vitamins and minerals, and different value-added products are produced [9]. Different parts of the date palm are used for different purposes: leaves are used for making roofs, mats, staple dishes, hand fans, baskets, packaging material, hats, ropes, fences and animal fodder [10]; trunks are used to construct houses, hives and bridges, and used as packing material [11]; terminal buds and young leaves can be cooked as vegetables, while rachises are used for paper making [12, 13]. Due to the extraordinary nature of the tree with its long-term productivity, the date palm tree is termed as the “tree of life”, “Bread of the Desert” [14] and the sacred tree [15].

Date palm is distributed throughout the Middle East, North Africa, South Sahel, areas of East and South Africa, and some parts of Europe and USA [2, 16, 17]. Date palm has been introduced to Ethiopia from Middle East countries approximately 200 years ago by Arabian traders [18]. Cultivation of date palm began in Afar region particularly at Afambo and Asayta and other places nearby Awash River and then spread to Errer Gota and Dredawa areas [19, 20]. Date farms of these areas are mainly used for local consumption and income sources through treading in nearby towns. In these areas, different unknown varieties of date fruits are produced which have red and yellow colours with different fruit shapes. In Ethiopia, around 14 known varieties have been introduced from other countries for the last 8 years and have been cultivated in three places: Humodoyta site (Afambo), Asayta and Melka Werer agricultural research centre (Melka Werer) for adaptation and improvement programme.

Evaluations of genetic diversity of date palm varieties at DNA level have great value in the date palm’s genetic improvement and conservation program. However, to date in Ethiopia, there was no research studied on genetic diversity and population structure of date palms. DNA typing has proven to be the most convenient method for screening variability between plant varieties, analysing genetic diversity and determining phylogenetic relationships among plants [17]. Many studies have been conducted to identify date palm genotypes using morphological traits and biochemical markers. However, using these markers alone, detection of genetic variation among genotypes is unreliable because these markers have been influenced by environmental factors and also show low levels of polymorphism [2, 17, 21]. Different DNA markers have been applied to analyse the genetic relationship of date palm cultivars in many countries such as in Egypt [22, 23], Tunisia [4, 24], Morocco [3], Nigeria [25], Pakistan [2] and Syria [26]. It is well known that microsatellite markers are still one of the most powerful molecular tools due to their nature and reproducibility for assessment of genetic diversity, population structure and differentiation. Therefore, the objective of the present research aimed to assess the genetic diversity and population structure within date palm genotypes collected on different locations and early introduced date palm varieties using microsatellite markers.

## Methods

### Sampling and DNA extraction

**Fig. 1:**
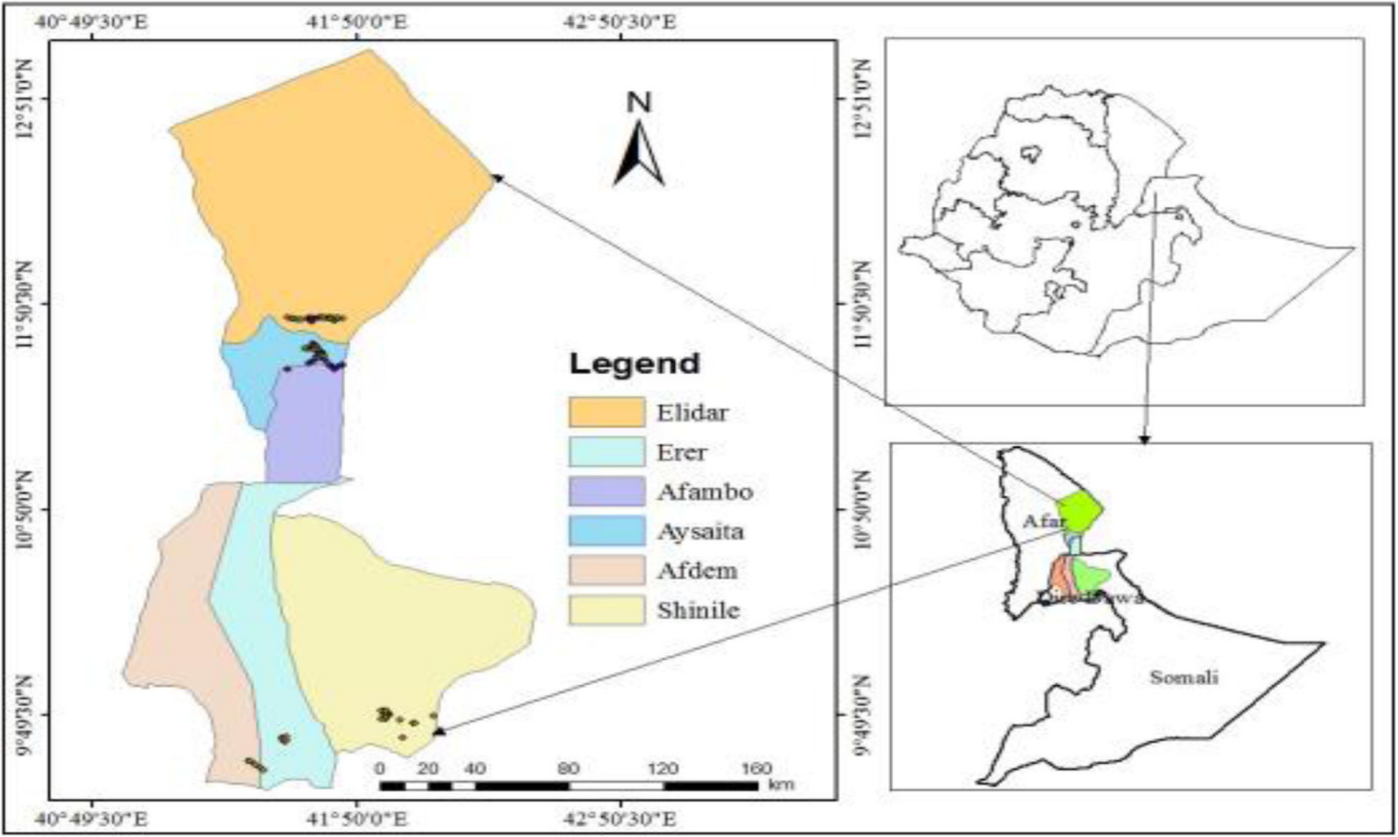
Geographical location and distribution date palm samples used in this study based on GPS system (black dots on map represent location of date palm trees)

During the time of fruiting, a total of 124 date palm samples both females and males that were collected in Afar and Somalia regions of Ethiopia (Fig. 1) which included early introduced date palm varieties collected from Afambo district specifically located at Humodoyta *Kebele* (in vitro date palm adaptation farm), and information related to data collection of all samples is available in detail as additional file with a manuscript (in Additional file 1 Table 1). Young and yellowish date palm leaf samples were collected from offshoots, cut into pieces and preserved in silica gel. The dried leaves were ground using liquid nitrogen and acidic sterilised sand. Genomic DNA was extracted using the CTAB method in 100 ml of CTAB buffer (pH 8.0) containing 2% of Cetyltrimethylammonium bromide, 4 ml of 20 mM EDTA, 4 ml of 100 mM Tris-HCl (pH 8.0), 8 g of 1.4 M NaCl and 0.1% β-mercaptoethanol. To test genome DNA quality, DNA (3 μl) was ran on 1% agarose gel using 0.5X TAE buffer, 90 V, 150 A, 50 W for 30 min set of the gel electrophoresis programme and stained with ethidium bromide solution and then illuminated under UV lights, and photographs were captured. DNA concentrations were also determined using a Gene Quant spectrophotometer.

### Polymerase chain reaction (PCR) amplification

A total of 10 simple sequence repeat (SSR) primers developed by [27] were used (Table 1) to amplify the isolated DNA. For PCR amplifications, 15-μl PCR reaction mixtures containing 100 ng DNA (1 μl), 10× PCR buffer (1.5 μl), 25 mM MgCl2 (1.2 μl), 10 mM dNTPs mixes (0.3 μl), 20 μΜ of forward and reverse primers (1 μl), 9.5 μl of PCR grade water, 5U Taq DNA polymerase (0.5 μl). All PCR amplifications were performed in thermal Cycler (Buio-Rad) with an initial denaturation step at 94 °C for 5 min and followed by 35 cycles denaturation at 94 °C for 30 s, annealing at 52 °C for 1 min, extension at 72 °C for 30 s, final extension at 72 °C for 5 min and hold period at 4 °C. The PCR products were separated using 1.5% agarose gels and stained in ethidium bromide solution and then illuminated under UV lights to be taken photographs. The 50 bp DNA ladder (Bio tools) was used to estimate the approximate molecular size of DNA fragments of PCR products (Additional file 4 Figure 1a-e).

**Table 1:** Microsatellite/SSR primers used for this study

No.	Primer name	Primer sequences	Repeated motif	Size range (bp)	Annealing temperature (°C)
1	MPdCIR010	F: ACCCCGGACGTGAGGTG R: CGTCGATCTCCTCCTTTGTCTC	(GA)22	142–162	52
2	MPdCIR015	F: AGCTGGCTCCTCCCTTCTTA R: GCTCGGTTGGACTTGTTCT	(GA)15	144–156	52
3	MPdCIR016	F: AGCGGGAAATGAAAAGGTAT R:ATGAAAACGTGCCAAATGTC	(GA)14	142–166	52
4	MPdCIR025	F:GCACGAGAAGGCTTATAGT R: CCCCTCATTAGGATTCTAC	(GA)22	220–272	52
5	MPdCIR032	F: CAAATCTTTGCCGTGAG R:GGTGTGGAGTAATCATGTAGTG	(GA)19	302–320	52
6	MPdCIR050	F: CTGCCATTTCTTCTGAC R: CACCATGCACAAAAATG	(GA)21	172–260	52
7	MPdCIR070	F: CAAGACCCAAGGCTAAC R: GGAGGTGGCTTTTGTAGTAT	(GA)17	200–250	52
8	MPdCIR057	F: AAGCAGCAGCCCTTCCGTAG R: GTTCTCACTCGCCCAAAAATAC	(GA)20	250–310	52
9	MPdCIR085	F: GAGAGAGGGTGGTGTTATT R: TTCATCCAGAACCACAGTA	(GA)29	166–244	52
10	MPdCIR093	F: CCATTTATCATTCCCTCTCTTG R: CTTGGTAGCTGCGTTTCTTG	(GA)16	175–227	52

### Data analysis

Amplified DNA fragments produced in each microsatellite locus was manually recorded (see Additional file 2 Table 2). Genetic diversity parameters, i.e. expected heterozygosity (He), observed heterozygosity (Ho), observed number of alleles (Na), effective number of alleles (Ne), private alleles per locus, Shannon information index (I) and Nm (Gene flow estimated from Fst = 0.25(1 − Fst)/Fst), were calculated by using Popgen32 version 1.31 [28] and GenAlEx version 6.5 [29] softwares. Major allele frequency (MAF) and number of genotypes (NG) per locus was performed using Power Marker software version 3.25. Polymorphic information content (PIC) of each locus was computed by Curves software version 3.0.7 and the fixation index (Fis, Fst, Fit) were calculated using Arlequin software version 3.5.2.2. Principal coordinate analysis (PCoA) was conducted from distance matrix of each accession using GenAlEx software. Ne’s genetic identity and distance was also performed by popgen32 software. Darwin software, version 6.0.21 was used to construct a dendrogram using the neighbour-joining (NJ) algorithm based on dissimilarity matrix and by computing bootstrap value over 1000 replicates. Population genetic structure was analysed based on Bayesian clustering using STRUCTURE 2.3.4 [30] to define the number of clusters in the dataset (i.e. ranging from *K*1 to *K*10). The admixture ancestry model and correlated allele frequency model were used to perform a Markov chain Monte Carlo simulation algorithm (MCMC). The length of the burn-in period was set to 100,000; MCMC after the burn-in period was set to 200,000, and was run 10 times for each *K* to estimate *K* values. Optimal *K* value among *K* groups was determined based on [31] the method using STRUCTURE HARVESTER [32] online website. The result files obtained from STRUCTURE HARVESTER was also analysed by CLUMPP software online website [33] to align the clusters across replicates and to display clusters in each *K* drawn as coloured box plots.

### Discriminant analysis of principal components (DAPC) and two-locus linkage disequilibrium (LD) analysis

In addition to STRUCTURE analysis, we performed DAPC, an assumption-free multivariate clustering method [34] using the R package ‘adegenet’ [35] to determine genetic structure date palm genotypes among districts. The optimal number of clusters was inferred using k-means analysis [35] of principal component (PC)-transformed SSR data, and Bayesian information criterion (BIC) was used to assess the best supported model (i.e. the number and nature of clusters). For the DAPC, eight clusters were chosen because they had the lowest value according to the BIC criterion, and 20 PCs were retained. We also calculated Ohta’s variance components of linkage disequilibrium [36] of D_IS_^2^ (the average disequilibrium within subpopulations), D_ST_^2^ (the contribution to the overall disequilibrium caused by differences in allele frequencies among subpopulations), D'_ST_^2^ (the variance of the correlation of linkage disequilibrium of one population relative to a total population) and D'_IS_^2^ (the variance of the correlation within population relative to that of the total population) using Popgen32 version 1.31.

## Results

### SSR marker and its allelic diversity

A wide range of allele variants were observed from each locus (Table 2). A total of 112 alleles were amplified with an average of 11.0 from all loci and a minimum (5.0) by MPdCIR032 and a maximum (16.0) number of alleles were revealed by MPdCIR085 and MPdCIR093 loci. The mean number of major allele frequency was 0.26, with numbers ranging from 0.155 (MPdCIR085) to 0.374 (MPdCIR016). All loci 307 genotypes were identified totally, and the number of genotypes per locus ranged from 7 (MPdCIR032) to 47.0 (MPdCIR085). The mean effective number of alleles was 6.61 ranging from 3.0 (for MPdCIR032) to 10.6 (for MPdCIR085). All SSR markers in this study was found to be highly informative with a PIC value ≥ 0.50 of which MPdCIR085 showed the highest PIC (0.899), while MPdCIR032 showed the lowest PIC (0.597) with an average of 8.09.

**Table 2:** Genetic diversity analysis of 10 polymorphic SSR markers for 124 date palm genotypes

Locus	MAF	NG	Na	Ne	Ho	He	Fis	Fst	Fit	Nm	PIC
MPdCIR010	0.183	40.0	12	7.94	0.669	0.874	0.209**	0.040*	0.241**	3.148	0.863
MPdCIR015	0.281	19.0	7	5.1	0.645	0.804	0.133*	0.891**	0.210**	2.057	0.776
MPdCIR016	0.374	29.0	11	4.94	0.508	0.798	0.356**	0.018	0.368**	3.443	0.779
MPdCIR025	0.297	30.0	13	6.3	0.355	0.84	0.548**	0.078**	0.584**	1.824	0.823
MPdCIR032	0.369	7.0	5	3	0.645	0.669	0.028	0.012	0.04	5.109	0.597
MPdCIR050	0.239	37.0	11	7.1	0.694	0.86	0.190**	0.01	0.198**	4.523	0.845
MPdCIR057	0.284	30.0	12	5.9	0.468	0.832	0.398**	0.079**	0.446**	1.637	0.812
MPdCIR070	0.304	27.0	9	5.6	0.694	0.822	0.165**	− 0.007	0.157**	6.432	0.803
MPdCIR085	0.155	47.0	16	10.6	0.71	0.906	0.194**	0.036	0.224**	3.273	0.899
MPdCIR093	0.179	41.0	16	9.45	0.726	0.894	0.178**	0.0018	0.194**	4.152	0.898
Mean	0.267	30.7	11.2	6.61	0.611	0.831	0.198	0.077	0.256	3.56	0.809
Sum		307.0	112								

Exact test significant at *P < 0.05, **P < 0.001

### Genetic diversity analysis

**Fig. 2:**
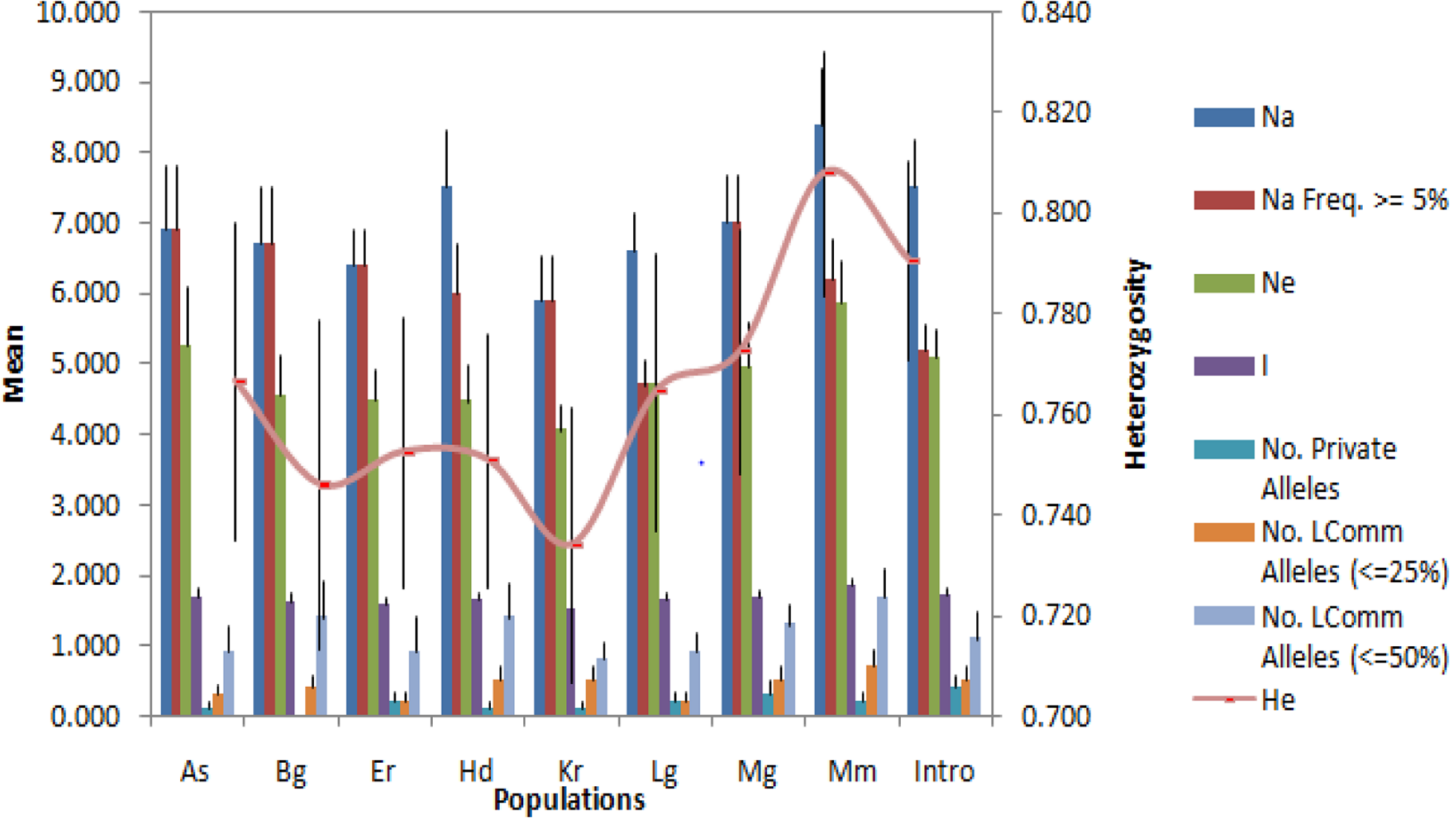
Allelic patterns across population of 124 date palm genotypes

In this study, high genetic diversity was shown among nine date palm accessions represented by a total of 124 genotypes. The genetic variation was estimated by observed heterozygosity (Ho), expected heterozygosity (He) and fixation index (Fis, Fst, Fit); these are presented in Table 2 and Fig. 2. The values of observed heterozygosity ranged from 0.355 for the locus MPdCIR025 to 0.726 for the locus MPdCIR093 with a mean value of 0.661. The highest expected heterozygosity value of 0.906 (for MPdIC085), lowest value of 0.699 (for MPdCIR032) and an average of 0.831 for all loci values were observed. In addition, across the population, high (0.81) and low (0.73) values of heterozygosity were observed in date palm populations collected in Mamulae and Kerebuda; also, the maximum mean numbers of alleles of date palm populations were 8.5 in Mamulae, 7.5 in Humodoyta and 7.5 in early introduced (Fig. 2). Although, in eight populations, a total of 16 private alleles were recorded, and no private alleles were seen in the Berga population (Fig. 2). Population differentiations were also determined by fixation indices (Fis, Fst, and Fit) for each locus with mean values of 0.198, 0.077 and 0.256, respectively. Fixation index (Fis) individuals relative to subpopulations ranged from 0.028 for locus MPdCIR032 to 0.548 for locus MPdCIR025, while Fst values ranged from − 0.007 (MPdCIR070) to 0.891 (MPdCIR015). From all loci except MPdCIR015, MPdCIR025 and MPdCIR057 showed moderate degree of genotypic differentiation between populations with Fst values of more than 0.05 with significant value at *P* < 0.001. The highest (7.0) and the lowest (4.7) mean values of the number of different alleles with a frequency ≥ 5% was in Mego and Legaharae, respectively. The mean of number of locally common alleles with a frequency ≥ 5% found in 25% of the total populations was ranged from 0.2 (in Errer Gota) to 0.7 (in Mamulae) whereas, the mean number of locally common alleles with a frequency ≥ 5% found in 50% of the total population ranged from 0.8 (in Kerebuda) to 1.7 (in Mamulae) (see Fig. 2). The degree of genetic similarity and distance between date palm populations are presented in Table 3. High genetic similarity was observed between Mamulae and Legaharae populations, while low similarity was between early introduced and Kerebuda. On the other hand, among populations we found lowest and highest genetic distance values 0.1791 and 0.6403 between Mamulae and Legaharae and Introduced and Kerebuda respectively. In addition, AMOVA showed that the prevalence of higher percentage genetic variation within individuals (73%) than among populations (4%) in Table 4.

**Table 3:** Nei’s genetic similarity (above diagonal) and genetic distance (below diagonal) between date palm populations

Pop ID	As	Bg	Er	Hd	Kr	Lg	Mg	Mm	Intro
As	****	0.7299	0.7543	0.7800	0.5838	0.7365	0.6259	0.7752	0.7476
Bg	0.3149	****	0.8165	0.7727	0.5800	0.7824	0.5990	0.7557	0.6696
Er	0.2819	0.2027	****	0.7478	0.5846	0.7661	0.5933	0.7242	0.6690
Hd	0.2485	0.2579	0.2906	****	0.5356	0.7281	0.6232	0.7434	0.7416
Kr	0.5382	0.5448	0.5369	0.6244	****	0.6347	0.7510	0.7795	0.5271
Lg	0.3058	0.2454	0.2664	0.3174	0.4546	****	0.6892	0.8360	0.6739
Mg	0.4686	0.5124	0.5220	0.4728	0.2863	0.3722	****	0.8007	0.5575
Mm	0.2546	0.2802	0.3227	0.2965	0.2491	0.1791	0.2222	****	0.7129
Intro	0.2909	0.4011	0.4020	0.2989	0.6403	0.3947	0.5843	0.3384	****

**Table 4:** Summary of AMOVA

Source	df	SS	MS	Est. Var.	%
Among populations	8	74.394	9.299	0.159	4%
Among individuals	115	575.037	5.000	0.968	23%
Within individuals	124	380.0	3.065	3.065	73%
Total	247	1029.431		4.191	100%

### Clustering and population genetic structure

**Fig. 3:**
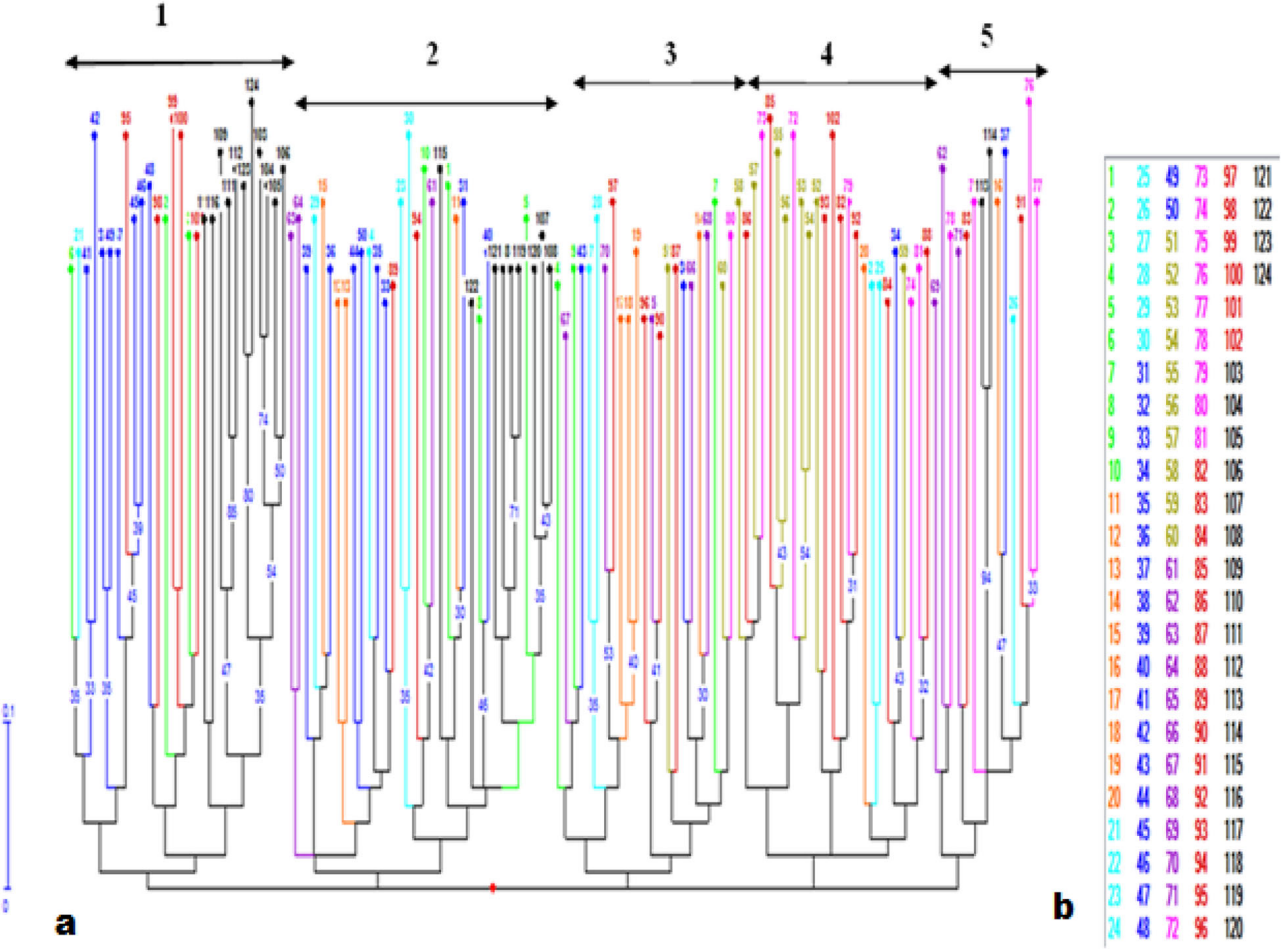
**a** A dendrogram representing the genetic relationship of 124 date palm genotypes based on dissimilarity matrix using NJ method indicated with bootstrap support ≥ 30 and **b** list of sample number for each population which are displayed on a tree based on colour labelling (i.e. 1–10 is the Alasabolo group, 11–20 is the Berga group, 21–30 is the Errer Gotta group, 31–50 is the Humedeyta group, 51–60 is the Kerebuda group, 61–71 is the Legaharae group, 72–81 is the Mego group, 82–102 is the Mamulae group and 103–124 is the Early introduced group

**Fig. 4:**
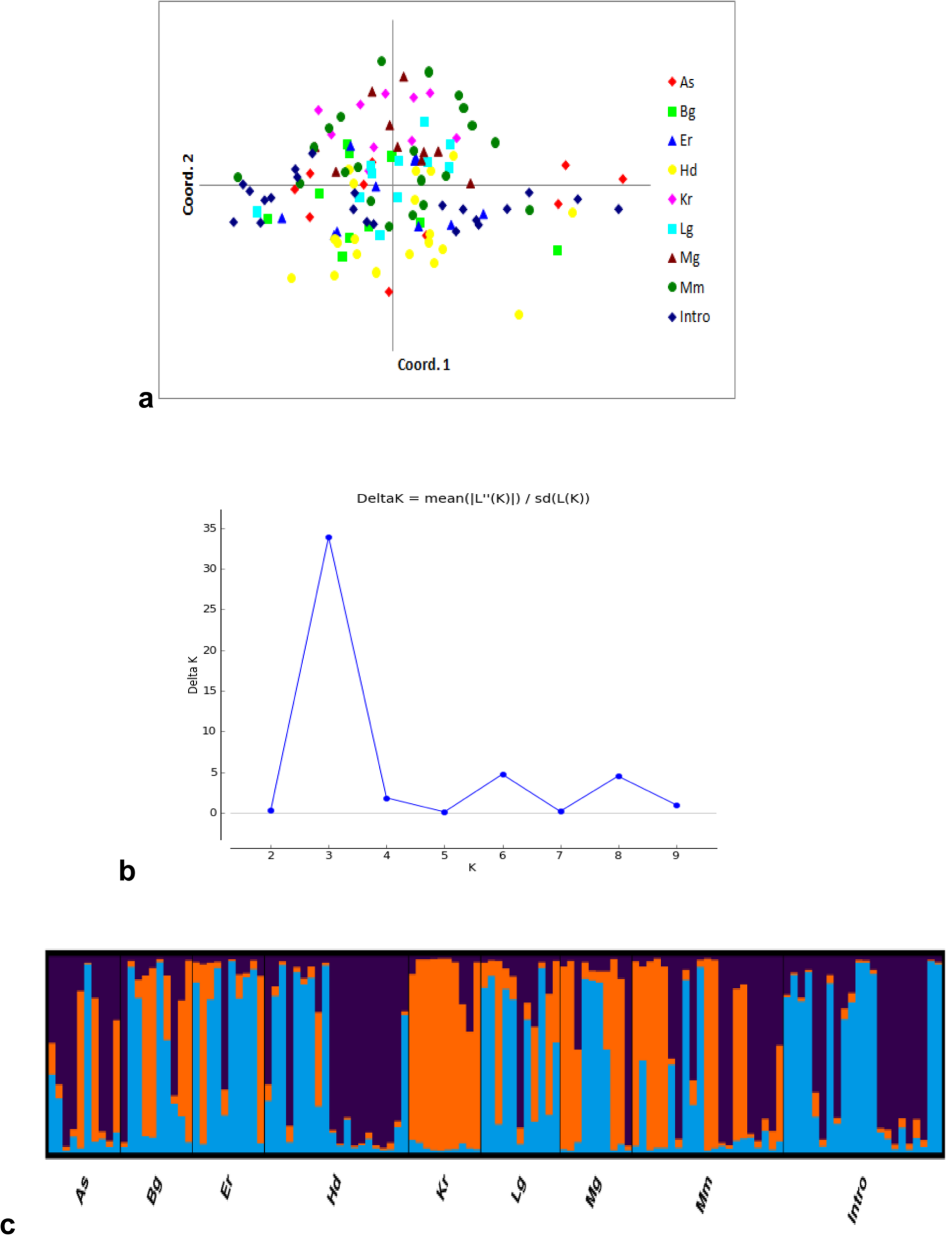
**a** Two-dimensional plot of principal coordinates analysis of 124 date palm genotypes that represent for nine populations. **b** Estimation of population using Δ***K*** = mean (|***L***”(***K***)|) / sd(***L***(***K***)) with cluster number (***K***) ranging from 1 to 10 and ***K*** = 3 is the optimal ***K*** value based on Evanno et al. (2005) method. **c** Results of STRUCTURE analysis based on microsatellite data and estimation of genetic structure of the nine populations using ***K*** = 3. Each population is represented by a vertical bar and separated by a black line, partitioned into coloured segments representing the proportion of the individual’s genome in the ***K*** clusters

A dendrogram and principal coordinate analysis were made to determine the structural similarity of among and within date palm populations and to predict the genetic difference between them. All genotypes separated into five major clusters (Fig. 3a), and one population is represented by the same colour as coded in the number list (Fig. 3b), and is similarly displayed in a dendrogram. In cluster 1, twenty eight individuals, cluster 2 thirty four, cluster 3 twenty three, cluster 4, twenty five and cluster 5 fourteen individuals were grouped as members. Clusters 1, 2, 3, and 5 were further subdivided into two subclusters, while cluster 4 was divided into three subclusters. All subclusters further subdivided into many subgroups which represented a clade composed of mixed genotypes of the populations. Half of the genotypes of each Introduced and Humodoyta populations were found together in cluster 1, whereas others clusters contained mixed genotypes from different populations. PCoA analysis revealed 17.33% total variation explained by the first 3 axes (Fig. 4a). The distribution of genotypes on the PCoA graph is not separately clustered depending on their accessions; instead, they showed intermixing of genotypes among populations and also similarly displayed on NJ tree. In population STRUCTURE analysis, date palm genotypes from all accessions were segregated into three subpopulations in the Bayesian clustering model using STRUCTURE software version 2.3.4 with the criterion of maximum membership probabilities based on [31] method, (i.e. the delta *K* value had the highest peak, at *K* = 3 (Fig. 4b)). The STRUCTURE output CLUMPP software online displayed light blue, orange and violet colours of bar plots (Fig. 4c) and revealed admixture structure in each date palm population regardless their accessions. The distributions of genotypes on the coordinate axis (Fig. 4a) as well as clustering of genotypes in a NJ tree (Fig. 3) were accord with the result in population STRUCTURE.

### Discriminate analysis of principal components and two-locus linkage disequilibrium

**Fig. 5:**
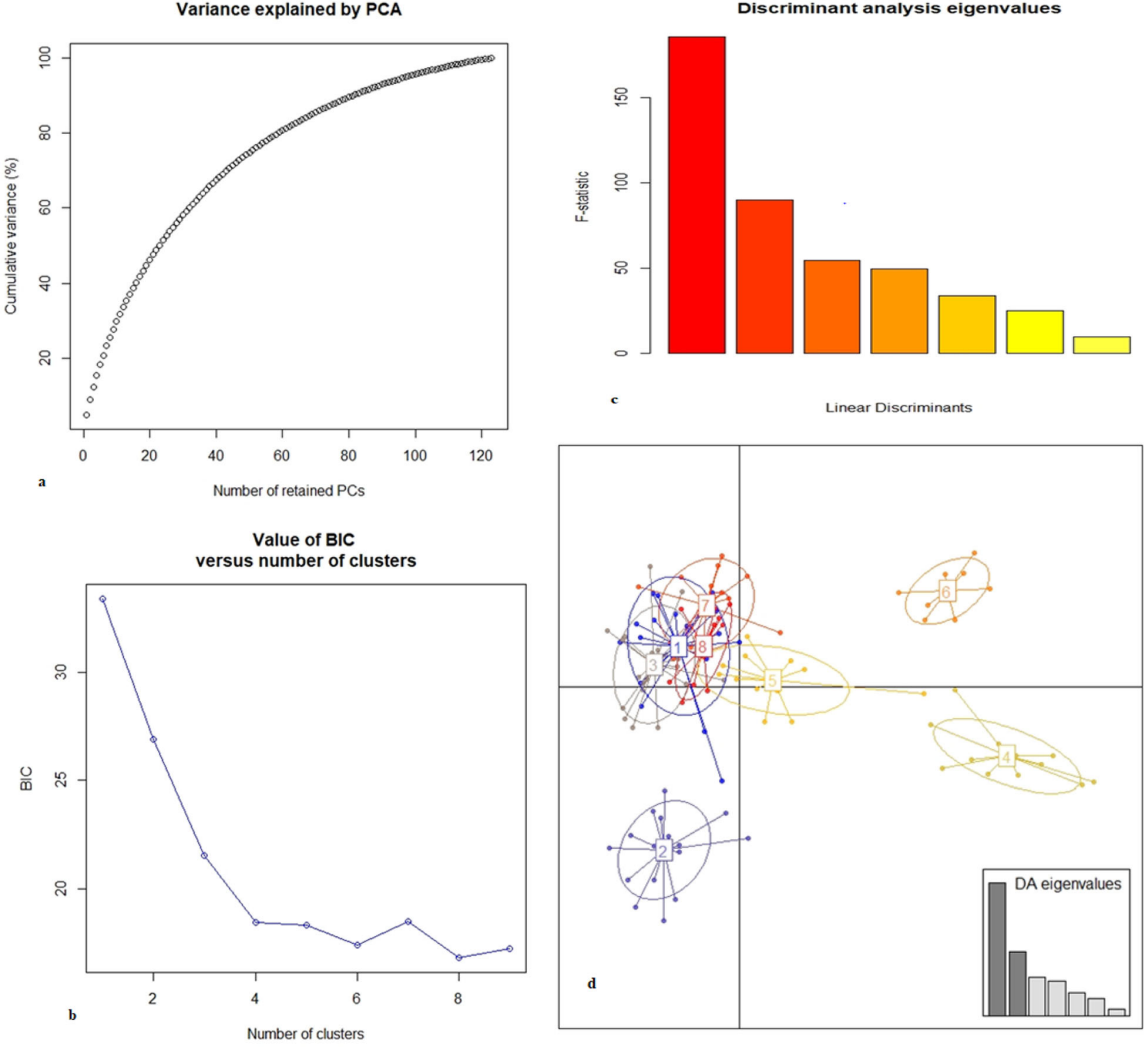
Bayesian model-based clustering of date palms among districts: **a** number of retained principal components, **b** value BIC and number of clusters, **c** discriminate analysis of eigenvalues, **d** discriminant analyses of principal components (DAPC) for 124 date palm genotypes collected from different districts. The axes represent the first two linear discriminants (LD); each circle represents a cluster, and each dot represents an individual. Numbers represent the different subpopulations identified by DAPC analysis

Using the Bayesian model-based approach, the membership probabilities of each date palm individual for the different groups were obtained from DAPC and the results of DAPC analysis. The DAPC separated date palm genotypes into eight clusters based on the first two linear discriminants (Fig. 5 and in Additional file 3 Table 3). The DAPC analyses revealed that all of early introduced date palm genotypes except one individual were represented by two genetic clusters (DAPC clusters 4 and 6). This result suggests that these date palm cultivars are genetically distinct groups from others. Besides, the NJ tree pattern showed that these introduced date palms are predominantly found in subcluster of the major clusters 1 and 2 of the NJ tree; this result is consistent with the DAPC clusters 4 and 6. The DAPC clusters 1 and 3 only contained 21 and 23 individuals from Afambo and Asayta district respectively, while the DAPC clusters 2, 5, 7 and 8 represented mixed individuals from all districts; i.e. DAPC cluster 2 contained a total of 18 individuals: 3 from Afambo, 12 from Asayta and 3 from Shinile; DAPC cluster 5 comprises a total of 13 genotypes: 3 from Afambo, 1 from Asayta, 1 from Errer, 7 from Shinile and 1 introduced cultivar and DAPC cluster 7 consisted 6 individuals from Afambo, 1 individual from Asayta, 9 individuals from Errer Gota; the DAPC cluster 8 included 7 genotypes from Afambo, 4 genotypes from Asayta and 1 genotype from Shinile districts. In general, the DAPC result showed that individuals were slightly admixed structure as compared with the population STRUCTURE and NJ results. A total of 45 two-locus pairs were analysed to estimate variance components of linkage disequilibrium. The overall average variance of the disequilibrium of the individual compared with the total populations D_IT_^2^ was 0.04681. A total average of D'_IS_^2^ (0.0435) was larger than D’ST^2^ (0.0033). Besides, the average value of D_ST_^2^ (0.0265) was greater than the average of D_IS_^2^ (0.0201). From the total pairs of loci, only 30 pairs of loci was shown in the dual relationships of D_ST_^2^ > D_IS_^2^ and D'_IS_^2^ > D’ST^2^ in Table 5. This relation in pairs of loci indicated that nonrandom association of the SSR alleles at particular variable loci was mainly caused by limited migration and random process or genetic drift [36, 37]. However, the rest of the 15 pairs of loci were shown in the dual relationships of D_ST_^2^ < D_IS_^2^ and D'_IS_^2^ < D’ST^2^; this is because gametes with favorable combinations of alleles would increase in every population [36, 37].

**Table 5:** Ohta's two-locus analysis of linkage disequilibrium of 10 SSR markers

Locus A–Locus B	(DIT)^**2**^	(DIS)^**2**^	(D'IS)^**2**^	(DST)^**2**^	(D'ST)^**2**^
MPdCIR010–MPdCIR015	0.04751	0.01856	0.04314	0.03147	0.00437
MPdCIR010–MPdCIR016	0.04730	0.02264	0.04363	0.02487	0.00367
MPdCIR010–MPdCIR025	0.05556	0.02375	0.05144	0.03162	0.00412
MPdCIR010–MPdCIR032	0.04203	0.01575	0.03886	0.02806	0.00317
MPdCIR010–MPdCIR050	0.04232	0.02251	0.03863	0.01832	0.00369
MPdCIR010–MPdCIR057	0.05966	0.02227	0.05514	0.03522	0.00452
MPdCIR010–MPdCIR070	0.04375	0.02477	0.03905	0.01906	0.00470
MPdCIR010–MPdCIR085	0.04356	0.02536	0.03866	0.01964	0.00490
MPdCIR010–MPdCIR093	0.03898	0.02103	0.03474	0.01743	0.00423
MPdCIR015–MPdCIR016	0.05277	0.01889	0.04908	0.03436	0.00369
MPdCIR015–MPdCIR025	0.06567	0.01806	0.06213	0.04474	0.00355
MPdCIR015–MPdCIR032	0.05207	0.01177	0.05071	0.03777	0.00136
MPdCIR015–MPdCIR050	0.04054	0.01585	0.03838	0.02464	0.00216
MPdCIR015–MPdCIR057	0.06444	0.02086	0.06164	0.04634	0.00280
MPdCIR015–MPdCIR070	0.04272	0.01720	0.04057	0.02553	0.00214
MPdCIR015–MPdCIR085	0.04669	0.01901	0.04294	0.02665	0.00375
MPdCIR015–MPdCIR093	0.04104	0.01604	0.03844	0.02402	0.00260
MPdCIR016–MPdCIR025	0.05557	0.02307	0.05120	0.03331	0.00437
MPdCIR016–MPdCIR032	0.03057	0.01013	0.02922	0.02532	0.00136
MPdCIR016–MPdCIR050	0.04109	0.02180	0.03874	0.01827	0.00235
MPdCIR016–MPdCIR057	0.05689	0.02261	0.05336	0.03698	0.00353
MPdCIR016–MPdCIR070	0.03739	0.02041	0.03304	0.01797	0.00435
MPdCIR016–MPdCIR085	0.04812	0.02370	0.04499	0.02179	0.00313
MPdCIR016–MPdCIR093	0.04131	0.02302	0.03758	0.01799	0.00373
MPdCIR025–MPdCIR032	0.05555	0.01314	0.05354	0.04143	0.00201
MPdCIR025–MPdCIR050	0.04742	0.01989	0.04459	0.02653	0.00283
MPdCIR025–MPdCIR057	0.07457	0.02512	0.06850	0.04683	0.00608
MPdCIR025–MPdCIR070	0.05081	0.02290	0.04671	0.02691	0.00411
MPdCIR025–MPdCIR085	0.05249	0.02404	0.04776	0.02734	0.00473
MPdCIR025–MPdCIR093	0.05400	0.02914	0.04803	0.02467	0.00597
MPdCIR032–MPdCIR050	0.02965	0.01014	0.02870	0.02053	0.00095
MPdCIR032–MPdCIR057	0.05998	0.01488	0.05680	0.04419	0.00317
MPdCIR032–MPdCIR070	0.02851	0.01240	0.02729	0.01746	0.00121
MPdCIR032–MPdCIR085	0.04159	0.01533	0.03965	0.02655	0.00194
MPdCIR032–MPdCIR093	0.03874	0.01532	0.03682	0.02203	0.00191
MPdCIR050–MPdCIR057	0.05406	0.02282	0.05089	0.02983	0.00317
MPdCIR050–MPdCIR070	0.03817	0.02197	0.03593	0.01418	0.00223
MPdCIR050–MPdCIR085	0.03777	0.02190	0.03560	0.01652	0.00218
MPdCIR050–MPdCIR093	0.03628	0.02347	0.02347	0.03318	0.00310
MPdCIR057–MPdCIR070	0.05421	0.02165	0.05067	0.03015	0.00353
MPdCIR057–MPdCIR085	0.05418	0.02473	0.04909	0.02969	0.00511
MPdCIR057–MPdCIR093	0.05341	0.02510	0.04830	0.02515	0.01443
MPdCIR070–MPdCIR085	0.03828	0.02089	0.03571	0.01675	0.00257
MPdCIR070–MPdCIR093	0.03174	0.01794	0.02913	0.01411	0.00261
MPdCIR085–MPdCIR093	0.03734	0.02203	0.03446	0.01512	0.00288
**Overall average**	0.04681	0.02009	0.04348	0.02648	0.00332

## Discussion

The purpose of the present study is to determine the genetic diversity and relationship between date palm genotypes and populations using SSR markers. All of the SSR primers tested in this study demonstrated PIC values from 0.509 to 0.899; these are considered to be highly informative markers and the occurrence of allele variations among populations; this is also supported by previous studies [38, 39]. A total of 112 alleles were detected from 124 date palm individual samples in populations. This exhibits the presence of high genetic diversity within date populations. In this study, number of alleles/ locus varied from 5 to 16 and maximum number of alleles (16) was amplified by MdPCIR085 and MdPCIR093 loci. According to [3], a maximum number of alleles (11) was detected by MdPCIR050; ranging between 4 and 11, whereas [40] reported a number of alleles ranging from 6 to 15 per locus and maximum (15) number of alleles amplified by primer MdPCIR015. The current study showed high genetic diversity among Ethiopian date palm populations may be due to high heterozygosity ( > 0.72).

In this study, NJ tree and principal coordinate analysis outcomes provided clear genetic relationship between unknown date palm varieties and early introduced cultivated date palm varieties. The dendrogram generated five major clusters. Generally, in this study, there was no clear separation among genotypes according to their geographical locations. In cluster one, eight individuals from Humodoyta *kebele*, five individuals from Mamulae *kebele*, three individuals from Alassabolo *kebele* and one individual from Errer Gota were clustered together with these early introduced cultivated date palm varieties (i.e. Sagaii, Jarvis, Khadrawy, Khalas, Medjool Israel and Khayra). Other cultivated varieties (Mdjool England, Shish, Zamli and Ashal Al Hassa) were found in cluster two with mixed individuals from Legaharae, Berga, Humodoyta, Error Gota, Alassabolo and Mamulae populations. Barhee cultivated variety was only found in cluster five incorporated with individuals from other populations. Half of date palm genotypes from Kerebuda population mainly observed in the subcluster of a clade four categories. This result was also supported with results in PCoA and population STRUCTURE analysis (i.e., intermixing distributions of genotypes on principal component axis and admixture structures were observed). The present result of PCoA showed certain match with the previous studies reported by [24, 41], contrary to the report by [38–40, 42]. According to the model-based clustering for genetic structure of date palm individuals, three genetically distinctive subpopulations were presented that were not formed in line with their collection places. The highest mean similarity score was estimated at *K* = 3, the most probable clustering of populations was observed at this *K* level and showed admixture structure among populations. This result displays the date palm populations that have a common genetic background and also they shared common alleles between them. The most differentiated population in this study was observed in Kerebuda population, which showed fewer admixtures than the other populations. Generally, the structure result had shown shared ancestry between unknown date palm genotypes and known date palm cultivars that were early introduced. The admixture result of the present study at *K* = 3 is in agreement with those reported by [39, 40, 43]. Similarly, [44] revealed three differentiated date palm memberships at *K* = 3 using different SSR as well as four date palm groups at *K* = 4 using SNPs. In both markers, they reported the admixture population structure of date palms at best *K* value of each cluster that was collected from different parts of the world. Intermixing clustering results of date palm genotypes in structure analysis of the present study to some extent coincides with the previous finding at five different *K* groups that were studied using different SSR primers [1, 41]. The DAPC results of this study exhibited genetic differentiation between groups while overlooking within-group variation and achieved the best discrimination of individuals into predefined groups. We confirmed maximum likelihood-based clustering results using DAPC method that is considered free of Hardy-Weinberg and linkage disequilibrium assumptions. The DAPC approach relies on discriminant functions that seek to maximize the diversity between clusters while minimizing within-cluster diversity [34]. Due to this reason, DAPC mostly assigned individuals to single clusters [34]. DAPC was suggested as an alternative method to identify and describe clusters of genetically related individuals by analysing complex genetic data and detecting admixed individuals by determining the probability that each individual belonged to each cluster [40, 45].

Overall, the AMOVA result of expected heterozygosity revealed high genetic differentiation within date palm genotypes and the outcome of the NJ tree; PCoA and structure analysis of this study are dominated by admixture structure patterns among populations. This might be due to the codominant nature of microsatellite markers contributed to have high allele’s variations per locus as well as high heterozygosity within date palm genotypes due to DNA slippage during the process of DNA replication. DNA slippage is the mutation of microsatellite length during enzymatic replication of microsatellite regions that are usually the result of insertion and deletion of repeats in DNA strands [46, 47]. On the other hand, date palms are dioecious and cross- and wind-pollinated plant species. This nature of the palms facilitates broadened genetic variations within date palm genotypes. In the case of cross-pollinating species, it is obvious that within plant populations, they maintain high levels of genetic diversity due to their breeding system [48–50]. Moreover, at date palm collection sites of the current study, there was no integrated management system related to propagation for date palms, i.e. the plants are like wilds (W. Ahmed, personal communication). Consequently, this situation could increase the probability of date palms breeding from seeds rather than offshoots. The date palms germinated by seeds have a dioecious nature, so an increase in the genetic distance between date palm genotypes is expected; also, low rate of gene flow within the population contributes to exhibited high genetic diversity within populations rather than among populations.

## Conclusions

Preliminary research on genetic diversity of plant species is the most essential issue for long-term plant improvement and conservation and development of mechanisms for reduction of plant vulnerability. SSR markers used in the present study revealed high genetic diversity within date palm genotypes and date palm populations. Therefore, this finding contributes input information in genetic relationship between known cultivated date palm varieties and unknown date palm varieties for improvement and conservation programmes. Generally, this study will be an eminent evidence and source of information on genetic diversity of date palms in Ethiopia to regional and international genbanks.

## Supplementary Information

**Additional file 1: Table 1** Data information of date palms in Ethiopia during the time of sample collection and cods of samples that were used for analysis.

**Additional file 2: Table 2** Microsatellite genotypic data of 124 date palm genotypes using 10 SSR markers.

**Additional file 3: Table 3** Proportion of individuals assigned in each DAPC cluster.

**Additional file 4: Figure 1** Examples of PCR products obtained by the following SSR primers: a) MPdCIR016, b) MPdCIR050, c) MPdCIR085 and d) MPdCIR093 e) M: 50 base pairs molecular weight markers used as a reference for scoring in this study.

## Data Availability

Information-related sample collection and GPS data (Supplementary Table 1) and microsatellite genotypic data (Supplementary Table 2) of date palms are available with a manuscript as additional files. During acceptance of this article, all data that support the finding of this study should be archived in figshare repository.

## Abbreviations

AMOVA: Analysis of molecular variance
CTAB: Cetyl trimethyl ammonium bromide
DNA: Deoxyribonucleic acid
dNTP: Deoxyribonucleotide triphosphate
DAPC: Discriminate analysis of principal components
He: Expected heterozygosity
Ho: Observed heterozygosity
I: Shannon information index
LD: Linkage disequilibrium
MAF: Major allele frequency
Na: Observed number of alleles
Ne: Effective number of alleles
NG: Number of genotype per locus
PCoA: Principal coordinate analysis
PCR: Polymerase chain reaction
PIC: Polymorphic information content
NJ: Neighbour-joining
SSR: Simple sequence repeat
