# Supplementary figures and images for: Genetic diversity and population structure of date palms (Phoenix dactylifera L.) in Ethiopia using microsatellite markers
Source: J Genet Eng Biotechnol. 2021 May 7;19:64. doi: 10.1186/s43141-021-00168-5 (PMC8105468; doi:10.1186/s43141-021-00168-5)

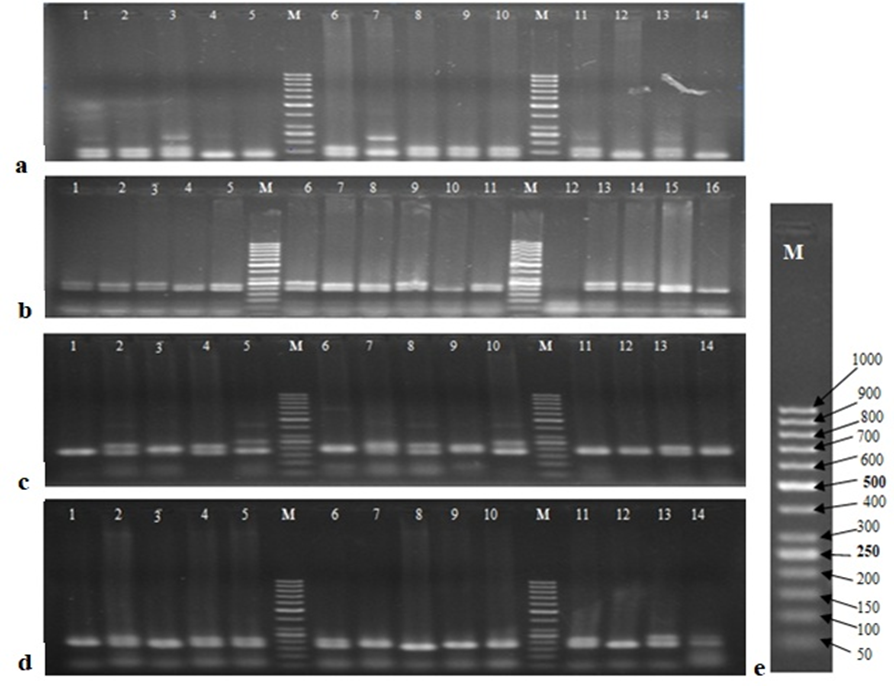


**Figure 1**

Supplement: Supplementary file 4 — Additional file 4: Figure 1 Examples of PCR products obtained by the following SSR primers: a) MPdCIR016, b) MPdCIR050, c) MPdCIR085 and d) MPdCIR093 e) M: 50 base pairs molecular weight markers used as a reference for scoring in this study. [file 43141_2021_168_MOESM4_ESM.docx]
